# Tetravalent Immunogen Assembled from Conserved Regions of HIV-1 and Delivered as mRNA Demonstrates Potent Preclinical T-Cell Immunogenicity and Breadth

**DOI:** 10.3390/vaccines8030360

**Published:** 2020-07-06

**Authors:** Nathifa Moyo, Edmund G. Wee, Bette Korber, Kapil Bahl, Samantha Falcone, Sunny Himansu, Adrianne L. Wong, Antu K. Dey, Mark Feinberg, Tomáš Hanke

**Affiliations:** 1 The Jenner Institute, University of Oxford, Oxford OX3 7DQ, UK; nathifa.moyo@ndm.ox.ac.uk (N.M.); edmund.wee@ndm.ox.ac.uk (E.G.W.); 2 Los Alamo National Laboratory, Theoretical Biology and Biophysics, Los Alamos, NM 87545, USA; btk@lanl.gov; 3 New Mexico Consortium, Los Alamos, NM 87545, USA; 4 Moderna Inc., Cambridge, MA 02139, USA; kapil.bahl@modernatx.com (K.B.); samantha.calabrese@modernatx.com (S.F.); sunny.himansu@modernatx.com (S.H.); 5 International AIDS Vaccine Initiative-New York, New York, NY 10004, USA; AWong@iavi.org (A.L.W.); ADey@iavi.org (A.K.D.); MFeinberg@iavi.org (M.F.); 6 Joint Research Center for Human Retrovirus Infection, Kumamoto University, Kumamoto 860-0811, Japan

**Keywords:** HIV vaccine, HIVconsvX, HIVconsv, conserved regions, T cell vaccine, mRNA vaccines

## Abstract

A vaccine will likely be one of the key tools for ending the HIV-1/AIDS epidemic by preventing HIV-1 spread within uninfected populations and achieving a cure for people living with HIV-1. The currently prevailing view of the vaccine field is to introduce protective antibodies, nevertheless, a vaccine to be effective may need to harness protective T cells. We postulated that focusing a T-cell response on the most vulnerable regions of the HIV-1 proteome while maximizing a perfect match between the vaccine and circulating viruses will control HIV-1 replication. We currently use a combination of replication-deficient simian (chimpanzee) adenovirus and poxvirus modified vaccinia virus Ankara to deliver bivalent conserved-mosaic immunogens to human volunteers. Here, we exploit the mRNA platform by designing tetravalent immunogens designated as HIVconsvM, and demonstrate that mRNA formulated in lipid nanoparticles induces potent, broad and polyfunctional T-cell responses in a pre-clinical model. These results support optimization and further development of this vaccine strategy in experimental medicine trials in humans.

## 1. Introduction

HIV-1 vaccine is needed as ever. Given the accumulating data on clinical benefits of HIV-1-positive individuals with T-cell responses targeting protective epitopes [1,2,3,4,5,6], selective pressure by T cells on the virus during acute and chronic HIV-1 infection [7,8], and several additional lines of evidence [9,10], vaccines to be effective may have to harness protective T-cell responses, if only to complement Env-induced antibody. To this end, we demonstrated in a series of phase 1 and 2 clinical trials induction of broadly specific T cells targeting conserved regions of HIV-1. These T cells inhibited viruses representative of four major clades and provided a signal of a durable virus control after stopping antiretroviral treatment (ART) in patients treated during primary HIV-1 infection [11,12,13,14]. Upgraded second-generation vaccine immunogens collectively called HIVconsvX with optimized conserved regions and increased match to global HIV-1 variants by a bivalent mosaic design [5] entered clinical evaluations in 2019. A strong correlation of high CD4 cell count and low plasma virus load with CD8^+^ T cells targeting the six regions of the HIVconsvX immunogen were observed in treatment-naïve naturally infected (not vaccinated) patients [4,6]. While in human studies, the HIVconsvX vaccines utilize a heterologous regimen of simian adenovirus prime-modified vaccinia virus Ankara (MVA) boost [5,15,16], we are relentlessly searching for alternative modalities of delivery to increase options for vaccine deployment [17,18]. Nascent mRNA technology is currently perhaps the most attractive novel platform. With a strong safety profile and potent efficacy demonstrated in animal models, mRNA has the potential to transform the field of human vaccines due to its numerous advantages including streamlined manufacturing, which in turn provides a possibility of greatly expedited iterative design optimization in humans [19,20]. In the present work, we evaluate preclinically a mosaic/epigraph mRNA vaccine delivering the second-generation tetravalent conserved regions of HIV-1 and demonstrate robust T-cell immunogenicity.

## 2. Materials and Methods

### 2.1. mRNA Synthesis and Lipid Nanoparticle Formulation

For each vaccine component, T7 RNA polymerase-mediated transcription was used in vitro to synthesize the mRNA from a linearized DNA template, which flanked the immunogen open-reading frames with the 5’ and 3’ untranslated regions and a poly-A tail, as described previously [21]. mRNA was then purified, diluted in citrate buffer to the desired concentration and encapsulated into lipid nanoparticles (LNP) by ethanol drop nanoprecipitation. At molar ratio of 50:10:38.5:1.5 (ionizable lipid:DSPC:cholesterol:PEG-lipid), lipids were dissolved in ethanol and combined with a 6.25-mM sodium acetate buffer (pH 5) containing mRNA at a ratio of 3:1 (aqueous:ethanol). Formulations were dialyzed against phosphate-buffered saline (pH 7.4) for at least 18 h, concentrated using Amicon ultracentrifugal filters (EMD Millipore, Temecula, CA, USA), passed through a 0.22-μm filter and stored at −20 °C until use. All formulations underwent quality control for particle size, RNA encapsulation, and endotoxin. LNP were between 80 and 100 nm in size, with >90% encapsulation of mRNA and <10 EU/mL of endotoxin.

### 2.2. Mice, Immunizations and Preparation of Splenocytes

Six-week-old female BALB/cJ mice were purchased from Charles River (Harlow, UK) and housed at the Functional Genomics Facility, University of Oxford. Groups of 6 mice were immunized intramuscularly with 3 µg for each vaccine component, delivering between 3 µg for monovalent and 12 µg for tetravalent vaccinations at weeks 0 and 4. Mice were bled at 1 week and culled at 5 weeks post boost. On the day of sacrifice, spleens were harvested and splenocytes were isolated individually by pressing organs through a 70 µm sterile nylon-mesh cell strainer (Fisher Scientific, Waltham, MA, USA) using a 5 mL syringe rubber plunger. Following the removal of red blood cells (RBC) with RBC Lysing Buffer Hybri-Max (Sigma-Aldrich, Pool, UK), splenocytes were washed and resuspended in R10 (RPMI 1640 supplemented with 10% fetal calf serum (FCS), penicillin and streptomycin, and β-mercaptoethanol for ELISPOT and intracellular cytokine staining (ICS) assays. All animal procedures and care were approved by the local Clinical Medicine Ethical Review Committee, University of Oxford and conformed strictly to the United Kingdom Home Office Guidelines under the Animals (Scientific Procedures) Act 1986. Experiments were conducted under project license 30/3387 held by T.H.

### 2.3. Peptides and Peptide Pools

The seven strongest H-2^d^ class I-restricted epitopes, previously well-defined in the BALB/cJ mice [17] and their variants present in the vaccine (Table 1), were used for immunological analyses. All peptides were >90% pure by mass spectrometry (Synpeptide, Shanghai, China) and were dissolved in DMSO (Sigma-Aldrich, Pool, UK) to yield a stock of 10 mg/mL and were stored at −80 °C.

### 2.4. INF-γ ELISPOT Assay

The ELISPOT assay was performed using the Mouse Interferon (IFN)-γ ELISpot kit (Mabtech, Nacka Strand, Sweden) according to the manufacturer’s instructions and as described previously [17]. Immune splenocytes were collected and tested separately from individual mice in triplicate wells. Peptides were used at 2 µg/mL each, and splenocytes at 2 × 10^5^ cells/well were added to 96-well high-protein-binding Immobilon-P membrane plates (Millipore) that had been precoated with 5 µg/mL anti-IFN-γ monoclonal antibody (mAb) AN18 (Mabtech, Stockholm, Sweden). The plates were incubated at 37 °C in 5% CO_2_ for 18 h and washed with phosphate-buffered saline (PBS) before the addition of 1 µg/mL biotinylated anti-IFN-γ mAb (Mabtech) at room temperature for 2 h. The plates were then washed with PBS, incubated with 1 µg/mL streptavidin-conjugated alkaline phosphatase (Mabtech) at room temperature for 1 h, washed with PBS, and individual spot-producing units (SFU) were detected as dark spots after a 10-min reaction with 5-bromo-4-chloro-3-idolyl phosphate and nitro blue tetrazolium using an alkaline–phosphatase–conjugate substrate (Bio-Rad, Richmond, CA, USA). SFUs were counted using the AID ELISpot Reader System (Autoimmun Diagnostika, Strassberg, Germany). The frequencies of responding cells were expressed as a number of SFU/10^6^ splenocytes after subtracting the no-peptide background frequencies.

### 2.5. Intracellular Cytokine Staining (ICS) Assay

Splenocytes or peripheral blood mononuclear cells (PBMC) were stimulated with peptide at 2 µg/mL; ionomycin and phorbol myristate acetate (PMA) at 2.0 mg/mL and 0.5 mg/mL, respectively, as positive assay controls; tissue culture medium with 1% DMSO was used as a negative control and processed as previously described [17]. The following mAb reagents were used: anti-CD107a phycoerythrin (PE)-conjugated mAb, anti-CD3 PerCP-eFluor710, anti-CD8a eFluor 450, anti-IFN-γ PE-Cy7, anti-IL-2 APC, and anti-tumor necrosis factor (TNF)-α fluorescein isothiocyanate (FITC) (all from eBioscience, San Diego, CA, USA) and anti-CD4 allophycocyanin (APC)/Cy7 (BioLegend, San Diego, CA, USA). Fixed cells were acquired on an LSRII flow cytometer (Becton Dickinson, Wokingham, UK). All mice were assayed in triplicates.

### 2.6. Statistical Analysis

Statistical analyses were performed using Graph Pad Prism version 7.0 (GraphPad Software, San Diego, CA, USA). For ELISPOT and ICS data, non-parametric tests were used and median (range) is shown. Multiple comparisons were performed using the Kruskal–Wallis test with Dunn’s multiple comparison post-test. Groups treated with the same in vitro stimuli were compared using two-tailed Mann–Whitney U tests. Two-tailed *p* values were used and *p* values of less than 0.05 were considered to be statistically significant.

## 3. Results

### 3.1. Tetravalent mRNA Vaccine HIVconsvM

**Figure 1:**
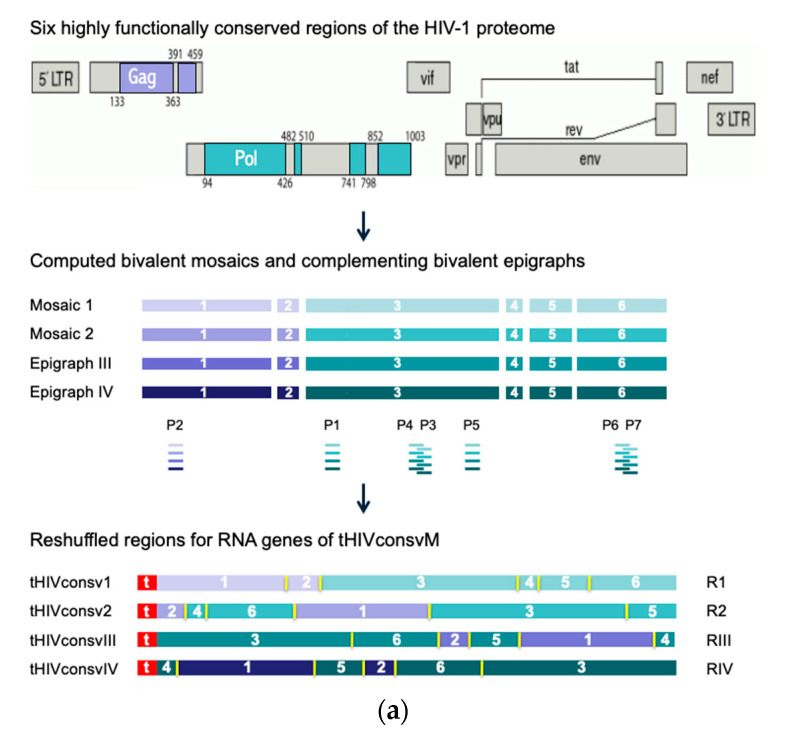

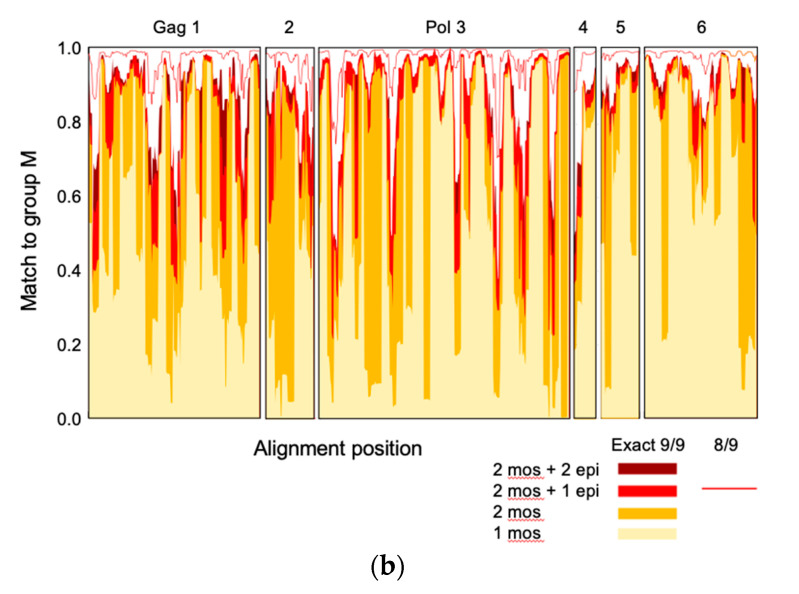
The HIVconsvM vaccine design. (**a**) Curated full-length-protein amino acid sequences of HIV-1 present in the Los Alamos National Laboratory HIV Molecular Immunology Database (LANL-HMID) were used to compute first two mosaics (September 2013) and then sequentially two complementing epigraphs (September 2017; 4925 Gag and 2703 Pol sequences) [22,25]. The same dataset as used for mosaics was used to select 6 highly conserved regions of the HIV-1 proteome, which were reshuffled into unique orders to minimize the chance of inducing strong T-cell responses to potential non-HIV-1 neoepitopes irrelevant for protection [5], which might have been generated by two juxtaposed regions [11]. Mosaics 1 and 2, and epigraphs III and IV (color-coded) differ in approximately 1 amino acid per epitope and together maximize the match of the vaccines to globally circulating HIV-1 isolates of group M. Small ‘t’ in front of the name indicates the presence of the human tissue plasminogen activator leader sequence [23]. The tetravalent immunogens were collectively called HIVconsvM and consisted of 4 mRNA molecules designated R1, R2, RIII or RIV. Pools P1 to P7 depicted under the regions indicate the approximate positions of the studied epitopes. (**b**) The 9-mer potential T-cell epitope coverage provided by the 6 vaccine regions is based on sliding window of 9 amino acids across the immunogen. Mosaics 1 and 2, being designed together, alternate the most common variants between themselves and the coverages of one (pale yellow) and both mosaics together (gold) are shown. The gain by adding epigraph III (red) and epigraphs IV (brown) into to the cocktail are also shown. Finally, the thin red line shows the fraction of 9-mers in each 9-mer window that matches 8/9 amino acids. Additionally, see Figure S1.

Previously, two Gag and four Pol protein regions totaling 872 amino acids, or 28% of the HIV-1 proteome, were selected for their high conservation among the HIV-1 group M isolates and inclusion of beneficial regions [2]. These were computed into two complementing mosaics, which together achieved over 80% match of potential 9-mer T-cell epitopes over most of the six HIVconsvX regions [5]. Here, using a novel epigraph algorithm [22], two versions of the same six regions were sequentially designed (Figure 1a) to cover additional common variants of potential 9-mer epitopes and make further improvements over the previous vaccine in matching the global HIV-1 isolates (Figure 1b). Using the mRNA platform, the human tissue plasminogen activator leader sequence was coupled to the start of the immunogen, which was shown to improve T-cell induction following intramuscular delivery of nucleic acid vaccines [23]. Four corresponding synthetic DNA fragments coding for the conserved regions attached to 5’ and 3’ untranslated regions and a poly-A tail were converted into mRNA using T7 RNA polymerase-mediated transcription, the cap structure was added at the 5’ end and the fully synthetic mRNAs designated R1, R2, RIII and RIV, and collectively called HIVconsvM (Figure 1a), were encapsulated into LNP as previously described [24].

### 3.2. mRNA Induces Strong and Broad T-Cell Responses

**Figure 2:**
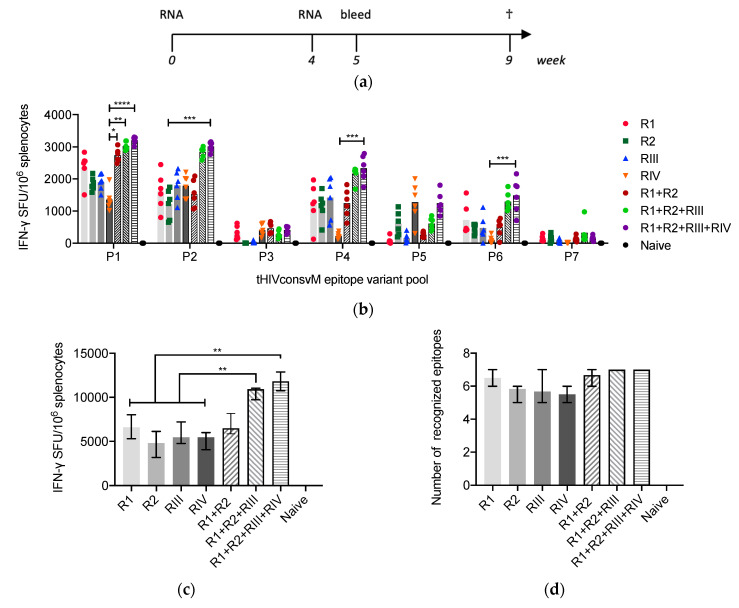
Frequency and breadth of CD8^+^ T cells induced by mRNA. (**a**) Groups of BALB/cJ mice received two intramuscular injections of mRNA as monovalent (R1, R2, RIII or RIV) and multivalent (R1 + R2, R1 + R2 + III or R1 + R2 + RIII + RIV) vaccines at weeks 0 and 4, and were culled at week 9 (†). Each mRNA dose was administered intramuscularly at a 3 µg dose resulting in a total of 6-, 9- and 12 µg amounts for the combined R1 + R2, R1 + R2 + RIII or R1 + R2 + RIII + RIV vaccines, respectively. (b) Vaccine-elicited T cells in the spleen 5 weeks after the last vaccination were enumerated in an IFN-γ ELISPOT assay using peptide pools P1 to P7 corresponding to the 7 most immunodominant H-2^d^ class I-restricted epitopes and their 4 variants present in the vaccine (Table 1). Data show pool-specific frequencies of responding T cells as group median (column) and individual mouse values (*n* = 6), except for naïve mice (*n* = 3). Groups were compared using Kruskal–Wallis test with Dunn’s multiple comparison correction. (c) The total magnitudes of responses were calculated as a sum of P1–P7 frequencies and are shown as group median ± IQR (*n* = 6) except for naïve mice (*n* = 3). Groups were compared using two-tailed Mann–Whitney U test. (d) Average number with range of recognized epitope variants for each group of vaccinated mice are depicted. (a and b) Significant two-tailed *p* values are indicated by asterisks: * *p* < 0.05; ** *p* < 0.01; *** *p* < 0.001 and **** *p* < 0.0001.

Groups of 6 BALB/cJ mice were vaccinated with single mRNA components (R1, R2, RIII or RIV) or in combination (R1+R2, R1+R2+RIII or R1+R2+RIII+RIV) twice 4 weeks apart intramuscularly, and their T-cell responses were analyzed 1 and 5 weeks later (Figure 2a). Both the RNA dose and timing of sampling were based on our previous experience with mRNA vaccines [17,21]. Responses to seven known well-defined CD8^+^ T-cell epitopes were readily detected in the IFN-γ ELISPOT assay 5 weeks after vaccination. Four variants of each epitope as present in the vaccine components were assembled into small pools P1 to P7 (Figure 1a, Table 1) and used for stimulation. While significant differences between groups were only rarely reached, there was an overall trend of a direct correlation between the magnitude of vaccine responses and valency of the vaccine cocktail both for individual epitopes (Figure 2b) and the overall ‘global’ response, which reached median (range) 11,821 (10,268–13,506) SFU/10^6^ HIVconsvM-specific splenocytes (Figure 2c) keeping in mind that the absolute mRNA dose increased with each component added. In addition, the average number of targeted epitopes increased (Figure 2d), which is likely contributed to by both the recognition of more epitope variants and the larger magnitude of the total response.

### 3.3. Kinetics and Quality of the HIVconsvM mRNA-Induced T-cell Responses

**Figure 3:**
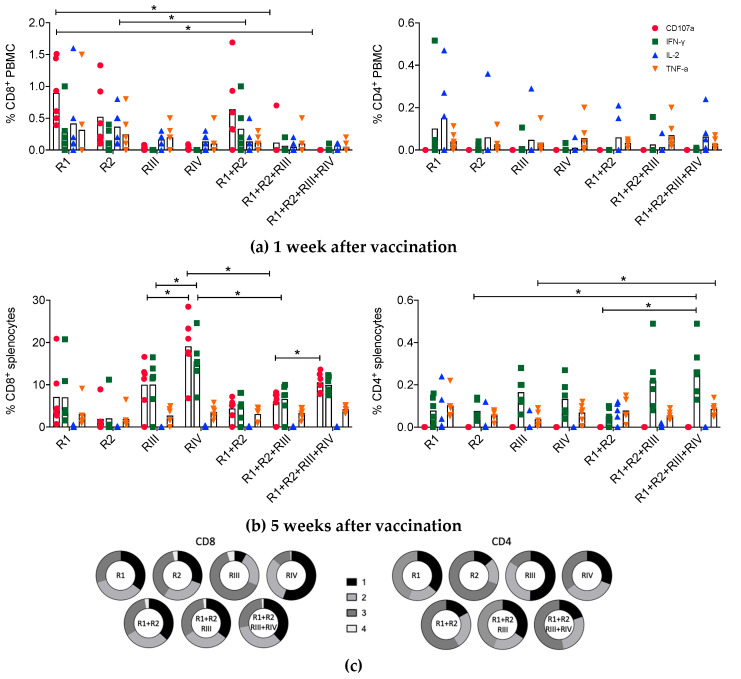
Functionality of mRNA-elicited CD4^+^ and CD8^+^ T cells. Groups of BALB/cJ mice were immunized with monovalent R1, R2, RIII and RIV or multivalent R1 + 2, R1 + R2 + RIII and R1 + R2 + RIII + RIV vaccines at weeks 0 and 4. Each mRNA was administered intramuscularly at a 3-µg dose resulting in a total of 6-, 9- and 12-µg amounts for the combined vaccines, respectively. The frequencies of responding PBMCs and/or splenocytes were determined using combined pool of 28 peptides corresponding to 7 defined H-2^d^ class I epitopes and their 4 variants present in the vaccine (Table 1). Multicolor flow cytometry analysis was performed to assess the functional phenotypes of vaccine-elicited CD8^+^ and CD4^+^ T cells in terms of CD107a, IFN-γ, IL-2 and TNF-α expression at 1 (**a**) and 5 (**b**) weeks after the homologous boost. The same cytokine legend as in (a) applies to (b). Specific T-cell frequencies are shown as group median (column) and individual animal values (*n* = 6). (**c**) Group median (*n* = 6) proportions of CD8^+^ and CD4^+^ T cells expressing 1 (black), 2 (light grey), 3 (dark grey) or 4 (white) functions are depicted using pie charts. See Figure S2 for the gating strategy. (a and b) Groups were compared using Kruskal–Wallis test with Dunn’s multiple comparison correction and significant two-tailed *p* values are indicated by an asterisk: * *p* < 0.05.

Polyfunctionality of mRNA-elicited CD8^+^ and CD4^+^ T cells was assessed at 1 and 5 weeks post-vaccination (Figure 2a). At 1 week after vaccination, specific peptides stimulated the expression of CD107a, IFN-γ, IL-2 and TNF-α in small fractions of murine PBMC populations reaching median 0.77% (CD107a) and 0.08% (IL-2) of CD8^+^ and CD4^+^ T cells, respectively (Figure 3a). At week 5 post-vaccination, broad, plurifunctional responses with peaks of median 19.3% (CD107a) and 0.26% (IFN-γ) in the CD8^+^ and CD4^+^ T-cell populations, respectively, were readily detected in mouse splenocytes (Figure 3b). Mostly, there is an overall good correlation between PBMC and splenocyte responses, although minor differences might sometimes occur. For CD8^+^ T cells, there were similar cell fractions that displayed 1, 2 and 3 functions with about 2% of tetrafunctional cells. CD4^+^ T cells induced by the full set of HIVconsvM mRNAs had similar proportions of cells with 1 and 2 functions, trifunctional cells represented about half of the population and there were no tetrafunctional cells (Figure 3c). CD4^+^ T-cells evaluation was likely an underestimation of true frequencies and functions, since optimal major histocompatibility complex (MHC) class I-restricted epitopes were used in vitro for restimulation of these MHC class II-restricted responses.

## 4. Discussion and Conclusions

In the present work, we tested an improved design of the second-generation conserved-region candidate T-cell HIV-1 vaccine by adding two frequent epitope variants and thus improving the coverage of global circulating HIV-1. In a mouse model, intramuscular injection of the LNP-encapsulated mRNA induced high frequencies of T cells, which were capable of recognizing seven out of seven examined H-2^d^ class I-restricted epitopes in most animals (Figure 2). All vaccine components were immunogenic individually and when combined, T-cell responses increased over 5 weeks post-vaccination, which concurred with our previous kinetic observations with another RNA vaccine platform [17]. Keeping in mind some experimental differences, the summed magnitude of T-cell responses reported here is superior to our published data for the bivalent HIVconsvX vaccine delivered using adjuvanted Semliki Forest virus-derived self-amplifying mRNA [17]. The reported frequencies induced by the bivalent R1 + R2 LNP vaccine were comparable to those elicited by a bivalent vaccine delivered by a heterologous simian adenovirus ChAdOx1 prime-poxvirus MVA boost regimen (N.M., E.G.W., B.K. and T.H., manuscript in preparation). Both these frequencies were surpassed by tri- and tetravalent R1+R2+RIII and R1+R2+RIII+RIV LNP immunizations (Figure 2c), which may be the result of the tetravalent design, the mRNA LNP delivery or their combination. In the BALB/cJ mice, we did not optimize the vaccine dose nor peak response time point. In addition, we did not test for the recognition of individual epitope variants as present in the tetravalent vaccine and those of other HIV-1 isolates listed in the LANL-HMID [26]. Nevertheless, our data show a great potency of this vaccine platform for induction of T-cell responses and support evaluation of the HIVconsvM mRNA LNPs in humans, the species in which HIV-1 evolved and for which further vaccine optimizations and response analyses will be the most relevant.

## Figures and Tables

**Table 1:** H-2^d^ epitopes and their frequent variants used for response detection.

Peptide Pools	Peptides	Vaccine Component
P1	VLVGPTPVNI	Mosaic 1
	VLIGPTPVNI	Mosaic 2
	VLVGPTPINI	Epigraph III
	VLVGPTPANI	Epigraph IV
P2	AMQMLKDTI	Mosaic 1
	AMQMLKETI	Mosaic 2
	AMQILKDTI	Epigraph III
	AMQILKETI	Epigraph IV
P3	IFQSSMTKI	Mosaic 1
	IFQCSMTKI	Mosaic 2
	IFQSSMTRI	Epigraph III
	IFQASMTKI	Epigraph IV
P4	SPAIFQSSM	Mosaic 1
	SPAIFQCSM	Mosaic 2
	SPAIFQASM	Epigraph III
	SPSIFQSSM	Epigraph IV
P5	REHLLKWGF	Mosaic 1
	RQHLLRWGF	Mosaic 2
	RAHLLSWGF	Epigraph III
	RQHLLKWGF	Epigraph IV
P6	ITKIQNFRVYY	Mosaic 1
	IIKIQNFRVYY	Mosaic 2
	IIKVQNFRVYF	Epigraph III
	ITKLQNFRVYY	Epigraph IV
P7	VYYRDSRDPI	Mosaic 1
	VYYRDSRDPL	Mosaic 2
	VYYRDNRDPL	Epigraph III
	VYFRDSRDPV	Epigraph IV

## Supplementary Materials

The following are available online at https://www.mdpi.com/2076-393X/8/3/360/s1, Figure S1: Coverage of 9-mers by the six second-generation vaccine conserved regions. Gradual improvements in the global HIV-1 coverage for the six conserved regions by increasing the valency of the vaccine cocktail form mosaic 1 (1), mosaic 1+2 (2), mosaic 1+2 + epigraph III (3) and mosaic 1+2 + epigraph III+IV (4) indicated on the x-axis. The fractions of 9-mers with an exact (red) and 8/9 (orange) matches are shown for each vaccine region separately, Figure S2: The ICS gating strategy. Gating strategy for identification of CD8^+^ and CD4^+^ T-cell functional phenotype within PBMCs and splenocytes.
