# Supplementary material for: Tetravalent Immunogen Assembled from Conserved Regions of HIV-1 and Delivered as mRNA Demonstrates Potent Preclinical T-Cell Immunogenicity and Breadth
Source: Vaccines (Basel). 2020 Jul 6;8(3):360. doi: 10.3390/vaccines8030360 (PMC7563622; doi:10.3390/vaccines8030360)
Supplement: Supplementary file 1 [file vaccines-08-00360-s001.pdf]

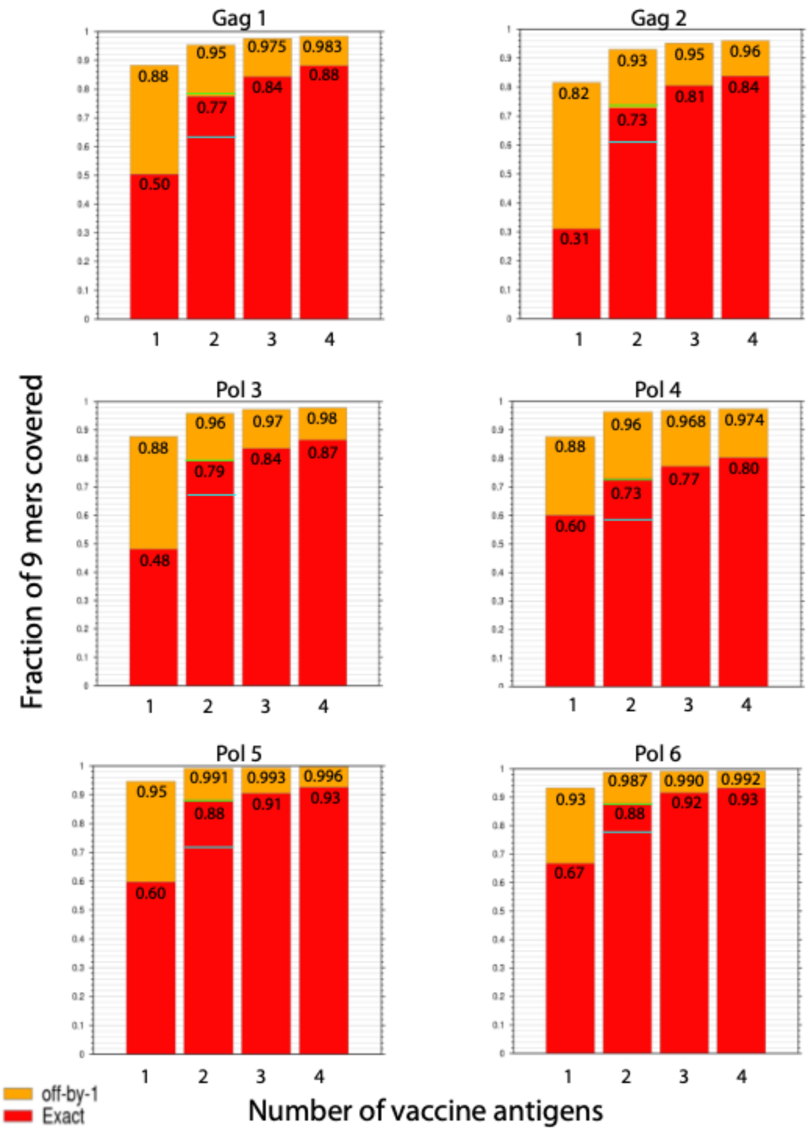

Figure S1. Coverage of 9-mers by the six second-generation vaccine conserved regions. Gradual improvements in the global HIV-1 coverage for the six conserved regions by increasing the valency of the vaccine cocktail form mosaic 1 (1), mosaic 1+2 (2), mosaic 1+2 + epigraph III (3) and mosaic 1+2 + epigraph III+IV (4) indicated on the x-axis. The fractions of 9-mers with an exact (red) and 8/9 (orange) matches are shown for each vaccine region separately.

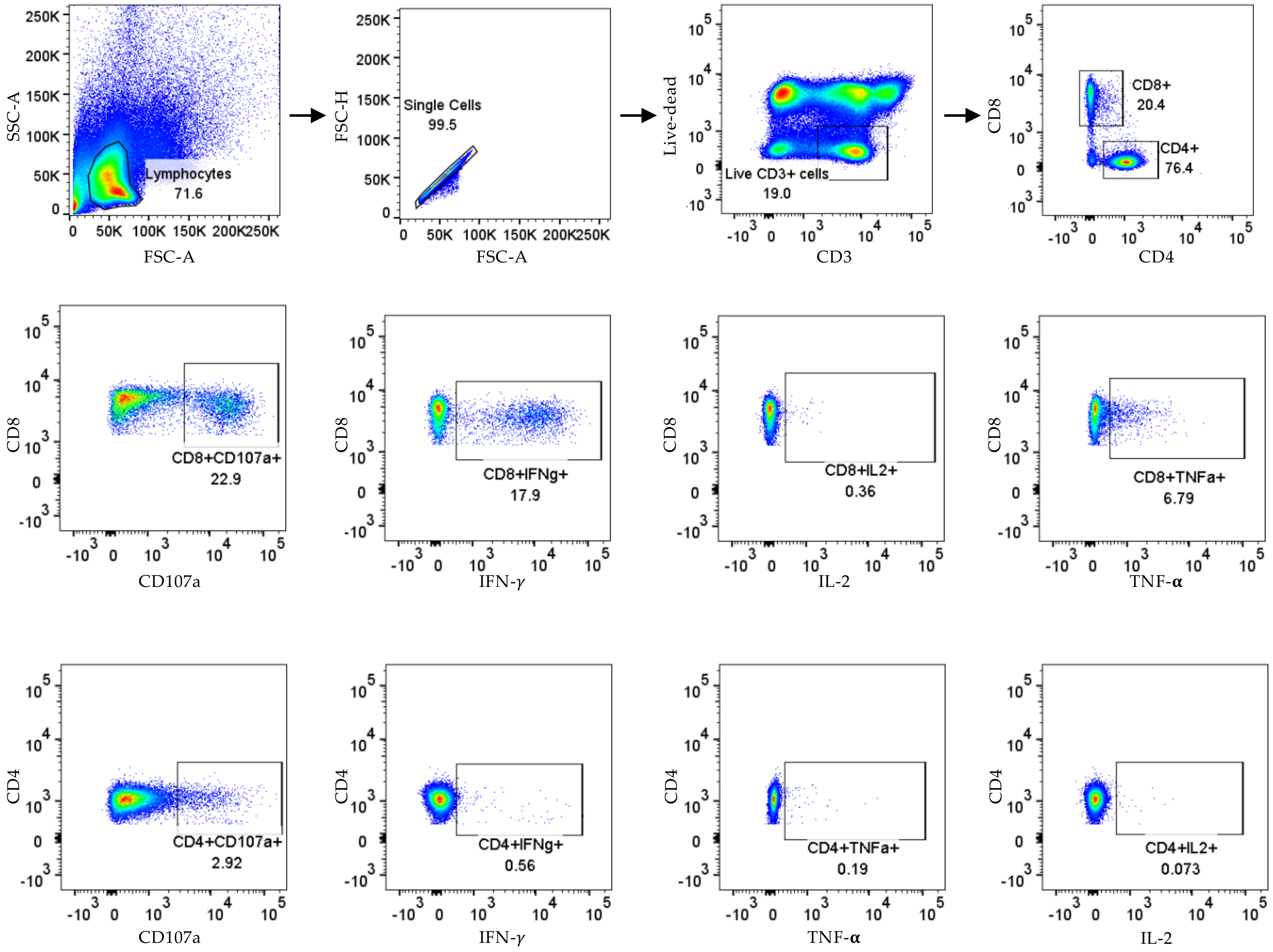

Figure S2. The ICS gating strategy. Gating strategy for identification of $CD8^{+}$ and $CD4^{+}$ T-cell functional phenotype within PBMCs and splenocytes.
